# Hospital and Institutional News

**Published:** 1920-04-17

**Authors:** 


### HOSPITAL AND INSTITUTIONAL NEWS

### HEALTH AND INDUSTRY.

It is reported that a definite proposal has now been put forward to found a National Institute of Psychology and Physiology, as applied to industry and commerce, and in the names representative of the powerful support which this proposal receives we find among the scientists and educationists, Dr. C. S. Myers, of Cambridge; Professor C. S. Sherrington, of Oxford; Professor E. H. Starling, of London; Sir Robert Blair; and Sir Alfred Keogh, and among those of the leaders of industry, Mr. W. L. Hitchens, of Messrs. Cammell, Laird and Co.; Sir Robert Hadfield; Mr. B. Seebohm Rowntree; and Mr. H. Gordon Selfridge. One of the first essentials to be considered will be the establishment of well-equipped laboratories for research into various occupations in order to determine the conditions necessary to get maximum output with minimum fatigue and discomfort to the worker. Innumerable problems call for immediate consideration, not in lengthy articles in the Press alone, educative though they may be, but the facts must be established and made available in readily understandable form. Further details of these proposals are given in our section on "The Public Well-Being" (p. 70).

### SMALLPOX AND VACCINATION.

We understand that the recent outbreak of smallpox in Glasgow has occasioned some anxiety to the health authorities concerned, in that many thousands of unvaccinated children may be found in the city. In one report we read that an exceptionally high mortality is occurring among those children already admitted to hospital. Our note on the value of vaccination is therefore opportune, and it cannot be too strongly urged that the evil influence of anti-vaccination agitators should be counteracted with all possible vigour.

### CANCER RESEARCH.

An important scheme at the Middlesex Hospital is now receiving official sanction from the Court of Governors. An amalgamation has been suggested between the Cancer Research Department of the hospital, the Bland Sutton Institute of Pathology, and the Middlesex Hospital Medical School, all of which stand upon the same site. The charge and centralisation of control will be largely formal, but the result will provide that close connection between the research laboratory and clinical sides of a medical establishment, the value of which process we have repeatedly urged in these pages. At the Middlesex the cancer research department has been established for over thirty years, and a cancer charity has been attached to the hospital since 1792. The Bland Sutton Institute is of more recent origin, but the medical school itself dates from 1774. From recent attention drawn to them, it is now well known that Chairs of Pathology, Physiology, Physics, and Radiology are also held at the hospital.

### RESEARCH DEFENCE.

We are glad to note that one at least of that section of the daily Press, whose comments upon matters medical we feel urged at times to criticise, is publishing a series of articles under the title of "Science and Practice," from which the public may generally learn what are the results achieved by recent research in medicine and surgery. Of late there have been so many innovations in official medical development and extension which cannot achieve their full purpose unless strongly supported by the general public. To mention but a few, there are the extended workings of local health authorities, the threatened restrictions upon animal experiments, the poverty of the voluntary hospitals, all of which are essentially in part dependent upon public approval or disapproval. Therefore we welcome such a straightforward yet simple exposition as comes from the pen of Mr. Stephen Paget on medical research and all it means to the nation.

### BIOGRAPHY OF SIR WILLIAM OSLER.

Lady Osler having requested Dr. Harvey Cushing, M.D., Surgeon-in-Chief of the Peter Bent Brigham Hospital, Boston, Massachusetts, to prepare a biography of the late Sir William Osler, Dr. Cushing will be most grateful for any letters or personal reminiscences, or for information concerning others who may possibly supply letters. Copies of all letters, no matter how brief, are requested, and if dates are omitted it is hoped that they may be supplied if possible. If the originals are forwarded for copy they will be promptly returned. All communications should be addressed to Dr. Harvey Cushing as above.

### OLDHAM INFIRMARY'S SECRETARY.

Mr. Charles D. Drake, late assistant-secretary of the Norfolk and Norwich Hospital, has been appointed secretary and superintendent of Oldham Infirmary in succession to Mr. E. L. Blake, and has already taken up his duties there. Mr. Drake was at Norwich for seven years, three of which were spent as assistant to Mr. Frank G. Hazell, the present general superintendent and secretary of Manchester Royal Infirmary.

### DEATH OF A GYNÆCOLOGIST.

Last week was announced in *The Times* the death of Professor Hector Treub, of Amsterdam University. Dr. Treub was, apart from being a well-known personality in the Netherlands, one of the most eminent gynæcologists in the country. He is perhaps better known in this country from his writings upon the early German invasion of Belgium and the scathing criticism to which he subjected our other enemies. Dr. Treub was born in 1856, and as a student at Leyden University, received his M.D. degree when only twenty-two years of age. We read that before his thirtieth year he had received two offers of a professorship, one for skin disease and the other for ophthalmology.

Obstetric work claimed his main attention, however, first at Leyden, and later in 1896, when he accepted the professorial chair at Amsterdam University on condition that a new clinic for women was established in that city. Both this and the Leyden clinics when under his control were well known in this country.

### DEATH OF A NOTED SCIENTIFIC ENGINEER.

A remarkable personality has passed away at Bournemouth in Mr. Charles Hearson, aged seventy-five years, the inventor of the well-known incubator. Mr. Hearson was a native of Barnstaple, Devon, and in early life was apprenticed to a chemist. Once he remarked that in those early days he made Ganot's "Physic" his Bible. He came to London over fifty years ago, and settled at Myatt's Park, Camberwell. He learnt engineering at South Kensington Science Museum, and after patient research and labour invented his incubators and foster-mothers, which have now a world-wide reputation. His keen insight turned his knowledge to use in surgical matters during the war. He applied the thermostat, an important feature of his incubators, to cupboards for sterilising surgical instruments, and the resulting appliances were used by Great Britain and the Allies. For many years he was the representative of Camberwell on the Metropolitan Water Board, where he devoted his energies to bacteriological research, which helped to ensure pure water to London. A man of many parts, he is widely missed in South London.

### GIFT TO COLLEGE OF SURGEONS.

At a quarterly meeting of the Council of the Royal College of Surgeons of England the following members of twenty years' standing were elected Fellows of the College: H. A. Thomson, C.M.G., M.D., C.M.Edin., surgeon to the Royal Infirmary and Professor of Surgery, University of Edinburgh, and W. T. Grenfell, C.M.G., M.D.Oxon., superintendent of the Labrador Medical Mission of Royal National Missions to Deep-sea Fishermen. At the same meeting a vote of thanks was given to Sir Rickman Godlee for presenting to the College the original drawings which he made for his "Atlas of Human Anatomy" in 1880, and also the calvaria of the patient whom he trephined in 1884 in order to remove a cortical tumour, thus performing the first operation of its kind.

### PENCILS AS CARRIERS.

We read that the finding of an inquiry instituted by the Ministry of Health when dealing with a recent diphtheria outbreak among children suggests that the infection has probably been conveyed from child to child by contaminated tops of pencils. It is a small but not unimportant point that the use of the same pencils by a number of children, or of pencils distributed among a class, collected, and redistributed another day, may be a potent means of disease conveyance if a carrier child be present. The biting of pencils is a bad and discouraged habit, but it is an optimist teacher who hopes completely to eradicate it from among a collection of juveniles. The lesson which this and other instances teach is that, as far as possible, not only pencils, but books and general desk equipment, should be distributed once and afterwards remain the particular property of the individual.

### POLICE STOP LOTTERIES.

Following upon the police action in respect of the "Golden Ballot" (during the adjournment of which case the promoters have taken the popular step of awarding the prizes) the police authorities have stopped a venture on a somewhat smaller scale in support of the West London Hospital. Some 200,000 tickets were issued at 6d. each, bearing the words: "This ticket is a receipt for a donation of 6d. for the West London Hospital." There were to have been prizes of one hundred guineas, fifty guineas, and twenty-five guineas, but the police have intervened, intimating plainly that the sweepstake must be stopped or a prosecution will result. It is a pity the police do not know their position in the matter of the legality of these ventures. It is said that they made inquiries in February in respect of the West London "sweep," and in March said it might proceed; now at the very last moment, just before the draw, they step in and cause a great deal of confusion. The hospital is willing to return the money to the subscribers, and has, in fact, done so in many cases, but we should not be surprised if the majority of people decide to leave their sixpences in the hands of those who will make the best possible use of them.

### ARSENIC IN SUGAR.

The outbreak of arsenical poisoning at Haslemere is reminiscent of the events of twenty years ago, when a large quantity of beer, distributed in the Manchester district, had been poisoned by the use of contaminated glucose, causing a number of deaths. The occurrence led to the appointment of a Royal Commission and to many improvements in the brewing trade, not only in the matter of the preparation of sugar substitutes, but in that of the roasting of malt, which formerly was sometimes done by means of coke, to the risk of the beer consumer. The present outbreak was confined to twenty households, and fortunately most of the sixty cases reported are light in character. The cause was quickly discovered to be sugar, and the local Medical Officer of Health, with a representative of the Ministry of Health, traced the supply to a shop where a barrel of moist sugar, recently arrived, had become contaminated on the railway by a liquid preparation for killing weeds, largely composed of arsenic. This occurrence has drawn attention once more to the dangers that may arise from foodstuffs and poisons being conveyed together on the railways.

### A WINDFALL FOR ST. DUNSTAN'S.

The ultimate residue of the estate of Mr. Francis Skey, of Maida Vale, W., has been left to St. Dunstan's Hostel for the Blind. The amount receivable by St. Dunstan's is expected to be about £20,000, and whenever it comes, very good use will be made of it, for the well-known institution for blinded soldiers and sailors has before it many years of activity not only in after-care work, but in present training, which is as extensive as ever owing chiefly to cases with injury to one eye becoming totally blind. St. Dunstan's is to be congratulated on the excellent fire-prevention arrangements at the hostel, which were tested by an actual occurrence of fire recently, happily quickly subdued. The completeness of the apparatus, its frequent testing and periodical drills of the blinded soldiers and nurses, under the superintendence of Messrs. Merryweather, are one testimony, if any were needed, of the thoroughness of the management of this institution.

### THE SUCCESS OF THE NOTTINGHAM APPEAL.

Two points deserve mention in the success which is attending, we may almost say has attended, the appeal on behalf of the Nottingham General Hospital. Mr. F. Acton, in a considered speech upon the report of the Hospital Saturday Committee, said that the appeal has gone well, and that the expenses of publicity has justified themselves. He also emphasised "the splendid response" which has rewarded the system of asking employers and employed jointly to agree to increase their efforts. The system has not been universally applied at present, but a personal request, on the lines so successful at Belfast, is to be made to each firm. The appeal, it will be remembered, included a request for an income of £20,000 a year. The miners' representatives have given a first-rate lead. They want their industry, from top to bottom, to bear its share; and the problem will be solved if other trades in the hospital area show equal promptitude and public spirit. It is believed that within a year the fund will show an increase of £15,000 from contributions, and that the pre-war total will be doubled. It is amusing to note that one place sent a deputation to ask what salary the Duke of Portland received for being president, and how much the chairman and the members of the board were paid. Mr. Acton replied reproachfully that, after twenty-six years' work for the hospital, he had not been "paid his screw yet; and, indeed, that they all worked for love and not for money!" This must have been an eye-opener to the deputation; but the question shows to what a pitch of commercialism we have come; and how necessary are the voluntary hospitals to set, and maintain, an uncommercial example.

### AN ADMIRABLE LETTER.

One of the most prettily worded appeals which it has been our good fortune to read is the letter written by Sir W. Treloar in the *Morning Post* of April 6. It is not for money, but for spades and pails to continue "the ambitious house, castle and town-planning scheme" which the crippled children of the Alton Hospital are carrying out on the beach at Hayling Island, where some of them are receiving "marine treatment." The children "fear no spite of wind or tide," and have an exponent of their wishes as imaginative as themselves. This admirable letter is as charming as it is brief. We offer it as a model to those who care for form as well as substance, and realise that the secret of style is a spontaneous sincerity in the writer.

### PATIENTS' PAYMENTS AT GLOUCESTER.

Colonel J. F. Curtis-Hayward, who presided at the recent annual meeting of Gloucestershire Royal Infirmary and Eye Institution, had some interesting things to say on the question of patients' payments. It appeared that the ordinary expenditure had increased by £2,162, and the ordinary income by £2,068. The chairman did not explain the relation of income to expenditure, nor how far these balanced at the end of the year. But he remarked that payments by patients had amounted to £3,226, an increase of nearly £2,000. The moral which he drew is that, while other hospitals are feeling the pinch, the Gloucester Royal Infirmary, thanks to the help of patients, has not yet felt it. This shows how experience of the plan differs in different localities; and though the instances of its success deserve to be recorded, there is no panacea in the system. In the main, as we have always contended, the burden of hospital finance must fall upon the healthy. Since the reserves of voluntary finance are inexhaustible (that is why voluntary hospitals cannot justly be called "insolvent"), the problem must be solved by the application of those methodical forms of collection which are congenial to this democratic age.

### LONDON'S PROSPEROUS HOSPITAL.

London has at least one prosperous hospital even at this moment, when the newspapers regard a prosperous hospital as something to be hushed up, so ill does it accord with the inviting gloom of their headlines. This enviable distinction belongs to the Poplar Hospital for Accidents, which the chairman, Sir Joseph Broodbank, lately reported to have finished the financial year "with £10,000 to the good." The festival dinner, at which this sum was asked for, produced £15,000. In this happy connection the name of Mr. Rogers, the secretary, deserves an honourable mention. Repairs and repainting are to be begun at once, and definite plans for rebuilding the west wing are being drawn. It is hoped that the present building prices will fall. We share the hope, but would like to know on what evidence the hospital relies for trusting to it. While subscriptions are slightly to the good, contributions have fallen from their once high place. Thus out of a total income, the largest ever received, of £28,791, a sum of £10,000 was placed to the credit of the building fund, which is now not far short of £30,000. We trust the situation means not a lucky year, but a good year, and that organisation will secure that the first fruits shall not be the whole harvest.

### A SUCCESSFUL APPEAL AT CHELMSFORD.

Last year was not supposed to be propitious for the issue of appeals, but when the Chelmsford and Essex Hospital asked for £20,000 to construct necessary extensions it received £18,000 and over in comparatively quick time. Two neighbouring houses have been purchased, and free right of way over the road between them and the hospital has been given. The houses will be used for the sisters and nurses, and thus add a hitherto occupied portion of the building to the wards, from which the waiting patients will benefit. A scheme based on the needs for the next ten years has been prepared, and it is hoped that money will continue to flow that it may be proceeded with. Miss A. M. Vipond, the secretary, has been congratulated on her work. Mr. E. E. Ridley is largely responsible for the success of the appeal, which again goes to show that existing sources of income can be developed, if method is applied to them. Conditions vary, but method generally means the organisation of support from regular bodies of subscribers, alike employers and employed. Such organisations not only bring money, but maintain an *esprit de corps* which is at once intelligent and reliable.

### OUR MEDICAL AMBASSADOR TO WASHINGTON.

Dr. S. D. Clippindale has made the appointment to the Embassy at Washington of Sir Auckland Geddes, M.D.Edin., the occasion of research into previous appointments of medical men to these posts. He informs *The Observer* of two instances. The first was the employment of Sir Thomas Elyot M.D.Oxon, author of the "Castell of Health," on diplomatic missions during the reign of Henry the Eighth. He deserves special mention, says Dr. Clippindale, because he was the first doctor to be knighted. The honour appears to have been given to Elyot for his diplomatic work. The second case recorded is that of Dr. Robert Jacob, M.D.Cantab, who "was sent by Queen Elizabeth to Russia to negotiate a marriage between Lady Mary Hastings and the Muscovite Prince, then in search of a seventh wife." The Prince's search, however, had exhausted his vitality, for he "died before arrangements could be completed." If these instances are complete, Sir Auckland Geddes is not the first medical man to hold the post of British Ambassador.

### THE SLUMP IN MANCHESTER.

Though Manchester confesses to be behind, even scandalously behind, other cities of equal pretension in support of its hospitals, which hitherto have appealed very largely in vain, there is no loss of faith in the voluntary system on the part of at least one chairman, Mr. Frank Roby, of the Manchester Children's Hospital. We find his faith invigorating, and congratulate him on it. Thus inspired, he and his colleagues should try to leaven the indifference of the wealthy city with a little method and organisation. Have they seen the Leeds scheme for an Employers' Subscription Fund; have they observed the example which Lady Pirrie has recorded of Belfast? There is no lack of ideas. The real want seems to be of men to put them to the proof of practice.

### A GREATLY OVERCROWDED STAFF.

A state of cramped administration prevails at Ancoats Hospital, Manchester; and since public sympathy has been aroused at the poor quarters which till lately have been the rule for nurses, is it too much to hope for a similar response on behalf of an administrative staff? Lord Cawley, the president, and Mr. William Armitage, the chairman, have jointly recorded the present situation as follows: "We are at the end of our resources. Our clinical staff is crowded into one small room which has served as a secretary's office for half a century. We have only one sitting-room for the combined use of our honorary and resident medical staff, and cannot offer a separate bedroom to each resident doctor." If such a state of affairs were revealed by a public inspection or inquiry it would be greeted as a scandal. But because it is pleaded in proof of the necessity of enlargement and as an undeniable claim to public support, nobody has paid much attention to it. Is overcrowding only bad for women? Do doctors suffer less from want of privacy than nurses? The idea is preposterous; and their needs should be the foremost ground of the appeal.

### LADY PIRRIE ON THOROUGHNESS.

We have already mentioned the success of the visits, on behalf of the Royal Victoria Hospital, Belfast, to the employers of the firms in the city, to which Lady Pirrie lately testified. In her still more recent address, which the audience desired to be printed (so full was it of spirit and ideas), Lady Pirrie proceeded to name the bodies from which she was hoping for support. The thoroughness of the list, within the scope chosen, is worth recording. She spoke first of the sportsmen of Belfast, and coupled with them the organisers of the city's amusements. She asked the theatres, halls, and picture-houses to allot one day a year to the hospital; the football associations to follow suit, "the wealthy and influential members" of the Belfast, Antrim and Down golf clubs to combine in a generous subscription. The Rotary Club, she hinted, had a scheme in hand, and the auctioneers and estate agents were uniting to form another. Thoroughness has already achieved much in Belfast, and belief in its power shows no sign of slackening. It is because such conviction is infectious that we note it here. What conviction can win in Belfast it can also win in London.

### THIS WEEK'S DRUG MARKET.

The market is still inclined to be quiet, but there is considerably more inquiry than there was last week, and, although there are signs of weakness here and there, prices, on the whole, are well maintained. Japanese refined camphor is noticeably lower in price. Japanese dementholised peppermint oil is quiet and lower. Cascara sagrada is cheaper. Lemon oil and bergamot oil have a lower price tendency. Citric acid continues scarce, and is again dearer. Makers of hypophosphites have advanced their quotations. Atropine is rather dearer. Castor oil is offered at lower rates. Potassium bromide is offered at lower rates. Cod-liver oil has a lower price tendency, and buyers are holding off. The value of opium is firmly maintained. Supplies of phenacetin are inadequate, and value has again moved upwards.

